# Empirical Evidence for the Rescue Effect from a Natural Microcosm

**DOI:** 10.3390/ani13121907

**Published:** 2023-06-07

**Authors:** Richard M. Lehtinen

**Affiliations:** Division of Reptiles and Amphibians, University of Michigan Museum of Zoology, Ann Arbor, MI 48109, USA; rlehtinen@wooster.edu

**Keywords:** *Guibemantis*, immigration, emigration, dispersal, population ecology, extinction risk, *Pandanus*, rescue effect, abandon-ship effect

## Abstract

**Simple Summary:**

A widely assumed ecological process called the “rescue effect” holds that populations of organisms that receive immigrants from other populations should be less vulnerable to local extinction than populations that do not receive such immigrants. While sensible, the rescue effect has had little strong support from real-world populations because of the difficulty of testing this idea. Using a study system of plant-specialist frogs from the rainforests of Madagascar, this study provides evidence for the rescue effect as well as weaker evidence for a related (but opposite) process referred to as the “abandon-ship effect”.

**Abstract:**

Ecological theory predicts that populations which receive immigrants are less vulnerable to extinction than those that do not receive immigrants (the “rescue effect”). A parallel but opposite process may also exist, where emigration increases the risk of local extinction (the “abandon-ship effect”). Using a natural microcosm of plant-specialist frogs from Madagascar, empirical evidence for both processes is provided. Populations receiving immigrants were less extinction-prone than those without immigration, and those populations losing individuals through emigration were more extinction-prone than those in which no emigration occurred. The number of immigrants and emigrants was also elevated and depressed (respectively) in patches that did not go extinct. These data provide some of the first definitive empirical evidence for the rescue effect and provide suggestive initial data on the abandon-ship effect. Both of these processes may be important to understanding the dynamics of populations.

## 1. Introduction

Small populations are widely thought to be more vulnerable to extinction from stochastic factors than larger populations [1,2]. Small populations can forestall extinction in two ways: by either successfully reproducing locally or by receiving immigrants from other populations. Either process increases the local population size, and this by itself reduces the immediate risk of local extinction. Immigration as a demographic mechanism that lowers extinction risk in small, spatially isolated populations (the “rescue effect”) was proposed almost 50 years ago by Brown and Kodric-Brown [3]. Island biogeography theory [4] provided the original theoretical context for the rescue effect and its intellectual descendent, metapopulation theory [5,6,7], also utilizes this concept. The rescue effect mechanism is also the logical basis for theories of source–sink dynamics [8] and the emphasis on corridors and population connectivity in conservation biology [9,10,11].

A related but different process called the genetic rescue effect has also been proposed which was originally defined as “the increase in the probability of a population’s survival due to the immigration of genes from another population” [12]. Since small, isolated populations can become inbred and lose genetic diversity via genetic drift over time, this infusion of alleles from other populations can increase subsequent offspring fitness and population growth rates [12]. The increased genetic variation from immigration can also lead to “evolutionary rescue” whereby small populations successfully adapt to a changing environment [13]. Both the genetic rescue effect and evolutionary rescue are now well-established phenomena, having been documented in varying degrees in a wide variety of organisms and circumstances [14]. However, empirical support for the original demographic rescue effect of Brown and Kodric-Brown [3] remains scarce.

If immigration can decrease the probability of extinction in small populations, the converse may also be true (i.e., emigration may increase the chance of local extinction). Since emigration necessarily reduces population size and smaller populations are more vulnerable to demographic and environmental stochasticity, emigration may have a negative effect on population persistence. Other researchers have informally recognized this parallel but opposite demographic process to the rescue effect [15,16,17]. This potential impact of emigration on extinction probability is referred to here as the “abandon-ship effect”. 

The theoretical bases for the demographic rescue and abandon-ship effects are clear and the mechanisms are plausible [3,18,19]. Nonetheless, robust empirical evidence from natural populations for these processes is minimal [17]. This relative lack of evidence does not necessarily imply that rescue and/or abandon-ship effects are absent in natural systems; rather, they may simply be empirically difficult to establish. At least three lines of evidence are necessary to document the operation of either process. First, individuals must be spatially segregated into semi-independent patches of habitat. Second, there must be at least periodic dispersal between these patches. Third, for the rescue effect, those patches that receive immigrants must have a lower extinction rate than patches that do not receive immigrants. For the abandon-ship effect, patches that lose emigrants must have a higher extinction rate than patches that did not lose emigrants. 

A wide variety of studies have satisfied the first two conditions by demonstrating movement between spatially segregated subpopulations [20,21]. Some studies have also presented genetic or demographic data indicating that immigration or emigration has some impact on the focal population [22,23,24,25,26,27]. However, dispersal is often inferred rather than directly measured, as it is often very challenging to distinguish between individuals born into a given population versus those that have immigrated there [17]. Further, extinctions often play out on considerable time scales (years to decades or longer [28]), necessitating empirical research that is long-term or spatially extensive (or both). Given these challenges, many studies in natural populations focus on one or a small number of study populations. However, to satisfy the third condition, it is not enough to show that immigration is associated with population persistence; one must also simultaneously demonstrate that lack of immigration decreases the likelihood of persistence, ideally in many replicate populations.

Due to the logistical difficulties of simultaneously assessing both inter-patch dispersal and patch extinction rates at large spatial (and often temporal) scales in replicate natural populations whose fate is initially unknown, the third condition has not yet been fully satisfied. Because of these real-world empirical challenges, demographic rescue effects have primarily been explored using computational models [29] and laboratory-based experimental populations [19,30,31,32]. Herein, empirical evidence is presented from a unique natural microcosm system that suggests that both the rescue effect and the abandon-ship effect influence extinction rates in a real-world population. Natural microcosms have the dual advantage of being more logistically tractable than systems at larger spatial scales; yet, there is no reduced level of biological realism [33].

## 2. Materials and Methods

### 2.1. Study System Description

At least eleven rainforest frogs in the genus *Guibemantis* (Anura: Mantellidae) from Madagascar are known to complete their entire life cycle in and on *Pandanus* plants (‘screw pines’, Pandanaceae; [34,35]). Some species of *Pandanus* retain rainwater in their leaf axils and these plant-held water bodies are used for breeding purposes in some *Guibemantis* [36]. In these species, eggs are laid on the surface of *Pandanus* leaves and, after a period of development, the hatchlings drop into the water-filled leaf axils [37]. After several months, aquatic tadpoles metamorphose into terrestrial juveniles [38]. Both juveniles and adults also remain on *Pandanus* plants and are found in no other microhabitats. *Guibemantis wattersoni* (the focal species in the present study) has a maximum life span of less than 12 months [39]. *Guibemantis wattersoni* was studied in a fragment of littoral rainforest near the village of Sainte Luce (Manafiafy) in southeastern Madagascar (24°46′ S, 47°10′ E, elevation 5–10 m asl). Other aspects of the natural history of this system are described elsewhere [40,41]. Note that older literature refers to *G. wattersoni* as *G. bicalcaratus* or *Mantidactylus bicalcaratus* (see [42]).

### 2.2. Field Surveys

**Figure 1:**
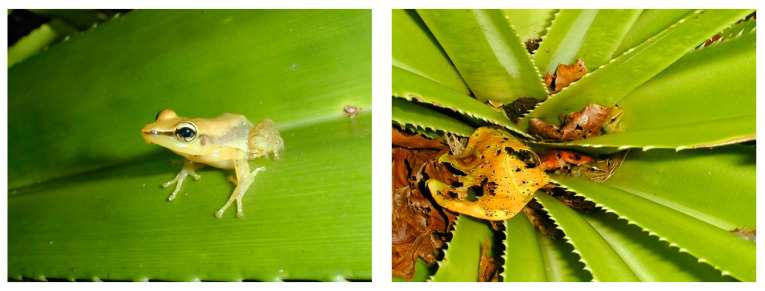
Adult *Guibemantis wattersoni* on a *Pandanus* leaf (**left**). *Pandanus* plant with three *G. wattersoni* (**right**). Photos by the author.

Because *G. wattersoni* completes its entire life cycle in the water-filled leaf axils of screw pine (*Pandanus*) plants (Figure 1), each plant is a patch of potentially suitable habitat. This system occurs on a relatively modest spatial scale; therefore, it was possible to simultaneously assess occurrence and abundance in a large number of patches as well as dispersal patterns among them. The spatial location of all *Pandanus* plants in two large networks of contiguous forest plots was mapped (total area: 18,750 m^2^), resulting in 839 mapped *Pandanus* patches. However, many of these plants were seedlings and not usable by *G. wattersoni*. Only considering *Pandanus* plants > 1.0 m in size in these two plot networks, 236 *Pandanus* patches were surveyed over a three-year period (2000–2002) to gather information on occupancy and turnover (local extinction and recolonization). The average distance between *Pandanus* plants occupied by *G. wattersoni* was 7.96 ± 4.28 m (network 1) and 8.60 ± 4.65 m (network 2; data from 2001). 

Three visual encounter surveys of each plant took place each rainy season between January and March (nine surveys overall for each plant throughout the study, 2124 plant surveys total). Surveys involved visually examining all leaves and leaf axils in each plant for any *G. wattersoni* individuals of any life stage. Searching continued as long as was necessary to be confident that no individuals were missed (usually less than ten minutes per plant). These small frogs are conspicuously colored, primarily diurnal and often active on the leaves of the plant [39]. This, combined with the fact that the plants are relatively small, resulted in high detection probabilities (see below). Most *Pandanus* plants were found on the forest floor; however, some ascended into the forest canopy. *Pandanus* plants whose height was greater than 1.5 m but less than 4.0 m were sampled using a stepstool constructed for this purpose. Plants higher than 4.0 m were sampled (when possible) by climbing adjacent trees using the single rope climbing technique. All field surveys were carried out by the author; therefore, inter-observer differences are not possible. The maximum number of *G. wattersoni* individuals detected in each plant in each year was used as a population size estimate in that plant for that year. Patches said to have experienced extinction were defined as those that were found to be occupied by at least one *G. wattersoni* individual in one year and were unoccupied in all surveys in the following year. Surveys were conducted between 0600 and 1700 h. Within years, each survey was separated by 14–20 days.

### 2.3. Mark-Recapture Study

A concurrent mark-recapture study in the same 236 patches was conducted to provide information on inter-patch emigration and immigration rates. Each rainy season, each patch was visited an additional four times within a 4-week period with each visit separated by 5–8 days (2832 visits total). These visits were in addition to, and separate from, the three annual visits to document occupancy and turnover. Individual frogs were hand-captured and marked by clipping toepads with sterilized scissors in unique combinations to allow individual recognition upon recapture [43]. Captured individuals were immediately released after marking. Since the two networks of plots were over 500 m away from one another (far beyond the dispersal ability of *G. wattersoni*), duplicate marks were used in each network to reduce the number of toepad clips needed to provide a unique identity. Clipped toepads of individuals from outside the immediate study area were clearly visible and showed no sign of regeneration after ten weeks. All captured adults and juveniles were toepad-clipped but recent metamorphs were not, as these were too small to safely capture and process. Together, these dispersal and turnover data enabled the direct comparison of patch-specific extinction rates with patch-specific dispersal patterns in the same time interval for a large number of populations. The mark-recapture study was used to document individual immigration and emigration but not to estimate population sizes, which was carried out with the field surveys (see above). 

### 2.4. Detection Probability

Empirical estimates of local extinction or recolonization can be influenced by detection probability during field surveys [18,44]. Specifically, changes in occupancy can be inferred erroneously if field surveys miss individuals that were actually present. Therefore, it is important to demonstrate that detection probability is relatively high to have confidence in local extinction and recolonization frequency estimates [45]. To estimate the probability of detection of *G. wattersoni* from the field surveys, presence–absence data from all surveys of all *Pandanus* plants carried out in all years was assembled. Using the program Presence (version 2.13.35; [46]), we ran a simple single-season occupancy model with no covariates to provide an estimate of the detection probability of the field surveys. 

### 2.5. Statistical Analysis

Fisher’s exact test was used to assess patterns in extinction rates among patches that did and did not receive immigrants or emigrants in the previous year. For comparison, a generalized linear model with binary logistic error structure was also run. This analysis used population persistence (yes or no) as the dependent variable and the number of immigrants and the number of emigrants as main effects. Student’s *t*-tests or Mann–Whitney tests were used to validate the assumption that small populations are more extinction-prone and to compare the number of immigrants and emigrants in patches in the previous year that did and did not go extinct. Statistical analyses were performed in IBM SPSS (version 28.0). Analyses only included patches that had both turnover and immigration and emigration information in each yearly time interval. *Pandanus* plants that were never occupied by *G. wattersoni* in any year were similarly excluded from the analyses.

## 3. Results

**Figure 2:**
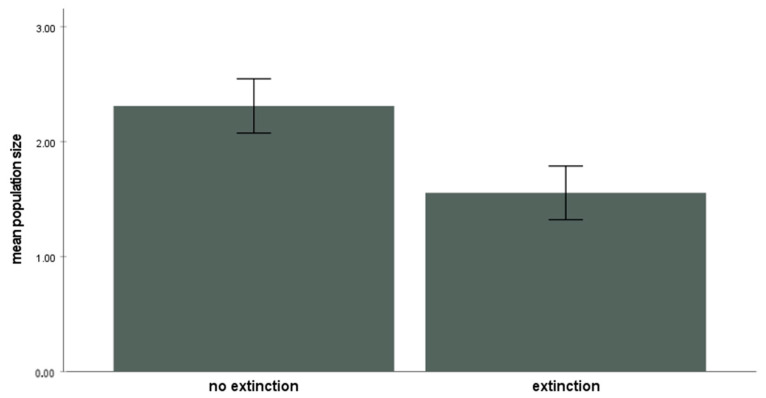
Patches experiencing extinction in a given year had a significantly smaller population size of *G. wattersoni* compared to patches that did not experience extinction (all years pooled, excluding unoccupied patches, Mann–Whitney test U = 9069.5, *n* = 380, *p* < 0.014). Error bars indicate means ± 2 SE.

*Guibemantis wattersoni* occupied an average of 63.4% (±9.7% SD) of the available patches (*Pandanus* plants) during the study period (range: 74.6% (in 2000) to 56.8% (in 2002). On average, 22.0% (±6.1%) of these occupied patches went extinct in any given year (*n* = 84 total). Those populations of *G. wattersoni* that went extinct were significantly smaller, on average, than those populations that did not go extinct (U = 9069.5, *n* = 380, *p* < 0.014; Figure 2). Population size per occupied plant ranged from 1 to 12 individuals (mean 3.48 ± 2.03 SD). 

A total of 567 *G. wattersoni* were marked in the mark-recapture study. Recapture rates were high within years (mean 53.8%, 305 recaptures total), but there were no inter-year recaptures. Dispersal among *Pandanus* patches was uncommon but not rare, with a mean of 13.9% (±6.8% SD) of marked individuals dispersing to a new patch per month. Mean dispersal distance for all known inter-patch movements was 15.5 m (±17.5 SD, range 0.5–123 m, *n* = 102).

In the 2000–2001 interval, dispersal data for *G. wattersoni* were available from 101 patches, 38 of which received at least one immigrant. Of the 38 populations which received immigrants, two went extinct in 2000–2001 (5.3%). In the remaining 63 patches (in which immigration was not detected) seven went extinct (11.1%; Fisher’s exact test, *p* = 0.477; Table 1). In 2001–2002, dispersal data were available for 89 patches, 39 of which received at least one immigrant. Two of these went extinct (5.1%). In the remaining 50 patches (with no immigration), four extinctions were recorded (8.0%; Fisher’s exact test, *p* = 0.692; Table 1). Pooling data for *G. wattersoni* from all years yielded dispersal and turnover information from 190 patches (77 with immigration, 113 without). The overall extinction rate in patches receiving immigrants was 5.2 ± 0.1% and the overall extinction rate in patches not receiving immigrants was 9.7 ± 2.2% (Fisher’s exact test, *p* = 0.288, Table 1). The number of immigrants per patch ranged from zero to four.

Of patches in which no *G. wattersoni* emigration was detected in 2000–2001 (*n* = 57), five went extinct (8.8%). In this same interval, five extinctions (11.4%) were recorded in patches where emigration was detected (*n* = 44; Fisher’s exact test, *p* = 0.744; Table 1). In 2001–2002, one extinction (1.9%) was recorded in those patches where emigration was not detected (*n* = 52). Among patches in which at least one emigration event was detected (*n* = 37), five extinctions (12.3%) were recorded (Fisher’s exact test, *p* = 0.078; Table 1). Pooled data for *G. wattersoni* from all years yields six extinctions (5.5% ± 4.9%) where emigration was not detected (*n* = 109) and ten extinctions (12.3 ± 1.5%) where emigration was detected (*n* = 81; Fisher’s exact test, *p* = 0.115; Table 1). The number of emigrants per patch varied from zero to three. Using data from all years, the generalized linear model did not find a significant effect of the number of immigrants or the number of emigrants on population persistence (likelihood ratio chi-square value = 9.17, df = 7, *p* = 0.241). 

**Figure 3:**
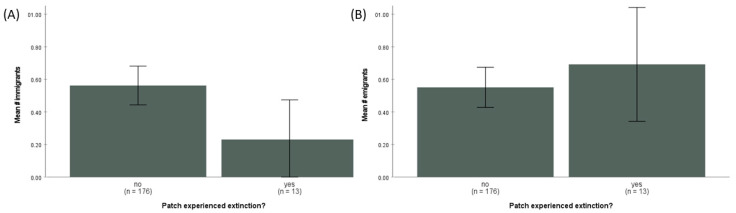
(**A**) Mean number (±2 SE) of immigrants to patches that went extinct (‘yes’) and those that did not experience extinction (‘no’), pooled data from all years 2000–2002. Patches that went extinct had significantly fewer immigrants (t = 2.45, df = 187, *p* = 0.025). (**B**) Mean number (±2 SE) of emigrants to patches that went extinct (‘yes’) and those that did not experience extinction (‘no’), pooled data from all years 2000–2002. Patches that went extinct had relatively more emigrants on average but this was not a significant difference (t = −0.607, df = 187, *p* = 0.544).

For patches that did not go extinct between 2000 and 2002, the mean number of *G. wattersoni* immigrants and emigrants per patch was very similar (0.56 ± 0.79 SD and 0.54 ± 0.81 SD, respectively, *n* = 175). However, patches that went extinct had significantly fewer immigrants (mean = 0.27 ± 0.46 SD; t = 2.45, df = 187, *p* = 0.025; Figure 3) and relatively more emigrants (mean = 0.73 ± 0.59 SD; t = −0.607, df = 187, *p* = 0.544; Figure 3). The single-season occupancy model confirmed that detection probability in the field surveys was high. The estimated detection probability of *G. wattersoni* in a single field survey was 0.806 (±0.011 SE; 95% C.I. 0.784–0.827).

## 4. Discussion

Using the three criteria from above to empirically demonstrate a demographic rescue effect or abandon-ship effect, this study has shown the following: (1) individuals of *G. wattersoni* were spatially segregated into semi-independent patches of habitat; (2) there was periodic dispersal between these patches; and (3) those patches that receive immigrants did have a lower extinction rate than patches that did not receive immigrants. For the abandon-ship effect, patches that lost emigrants had a higher extinction rate than patches that did not lose emigrants. While these differences do not quite reach statistical significance, the trends are uniformly in the expected directions (Table 1). Further, the expectation that smaller populations are significantly more extinction-prone was confirmed (Figure 2) and, importantly, patches experiencing extinction had significantly fewer immigrants (Figure 3). However, patches experiencing extinction did not have significantly more emigrants than patches not experiencing extinction (Figure 3). Thus, these results are in accordance with previous theory and predictions regarding the demographic rescue effect [3] and there is some intriguing suggestion of the abandon-ship effect as well.

Previously, the best empirical data from natural populations on the rescue effect were from the Glanville Fritillary butterfly [18]. In this famous study, more connected patches (as measured using the S_i_ metric) experienced significantly fewer extinction events, at least in smaller populations [17]. However, even in the Glanville Fritillary butterfly dataset, the evidence for the rescue effect was only correlative, since inter-patch movement was not measured directly but rather only indirectly inferred via relationships between patch size, patch isolation and dispersal behavior [18]. In the current study, because of the smaller spatial scale of the study system, direct measurement of immigration and emigration was possible. These observations show a clear trend for patches receiving immigrants to be less extinction-prone than those that did not receive immigrants (Figure 3; Table 1). While many variables such individual-specific (e.g., age or sex) or patch-specific characteristics (e.g., patch quality) may influence dispersal decisions, the over-arching result is that the arrival of immigrants tends to reduce extinction risk, regardless of the proximate reasons for their arrival. 

It is also notable that based on the estimated detection probability from the occupancy model results, the estimates of local extinction and recolonization frequency in this system seem reliable. Specifically, the chance of recording a *Pandanus* plant as unoccupied by *G. wattersoni* from a single field survey, when in fact it was occupied, was estimated using the model to be 0.194. With three surveys per plant per year, the estimated chance of mischaracterizing an occupied plant as unoccupied is less than 1% (0.194 × 0.194 × 0.194 = 0.0073). Thus, sampling error appears to be relatively unimportant in establishing the annual occupancy status of each plant and the resulting turnover estimates.

In this system, immigration reduces local extinction risk rather than increasing it through disease transmission or breaking up local adaptations (the “anti-rescue effect” [47]). The observed rescue effect is also very likely to be a demographic one (as originally envisioned by [3]) and not genetic. A genetic-rescue effect can occur when immigrants to small, genetically homogeneous populations rescue these populations from extinction by increasing subsequent genetic diversity [14,48]. The processes that result in low genetic diversity (e.g., inbreeding and genetic drift), however, can take many generations to accumulate [12]. The current study system, in contrast, is characterized by moderately low spatial isolation, substantial rates of inter-patch dispersal and rapid turnover dynamics [41], which make it very unlikely that small local populations are isolated enough or would survive long enough for severe inbreeding or genetic drift to be a problem.

Most population ecological models assume that there is either no correlation [49] or a negative relationship [8] between emigration rate and extinction risk. Alternatively, extinction probability could increase as the emigration rate increases if these departing individuals are not replaced by immigration or local recruitment. In these situations, small populations are likely to go extinct deterministically due to high emigration rates rather than as a result of demographic or environmental stochasticity. Several studies have provided some evidence that extinction is more probable as the number of individuals leaving the patch increases [16,50]; however, again, this is challenging to rigorously assess at large spatial scales in natural systems. The data presented here indicate a trend for extinction rates to be higher in patches with emigration compared to those without emigration (Table 1). However, while the number of emigrants tended to be higher in patches where extinction occurred compared to those that did not experience extinction, this was not a significant difference (Figure 3). Therefore, some of the evidence suggests that emigration by *G. wattersoni* increases the probability of local extinction; however, as of yet, this first test of the abandon-ship effect is only partially supported.

Patch occupancy in *G. wattersoni* varied over space and time during this study but was driven primarily by variation in patch height, patch size and patch quality [see [41] for full details]. The overall metapopulation of *G. wattersoni* was dynamic (approximately 22% of patches went locally extinct per year) but was fairly stable over the three years of the study. Thus, while local extinction was reasonably common, this was largely balanced by colonization. Additionally, as shown above, extinctions were made less likely by rescue from immigrants from other patches. 

## 5. Conclusions

Many phenomena in ecology are difficult to address empirically in natural systems [51]. Nevertheless, finding ways to test theory with empirical data is critical to further our understanding of how the natural world works. The unique features of this natural microcosm from the rainforests of Madagascar permitted one such test of an important and widely assumed ecological process (the rescue effect). These data suggest that, in this system, the rescue effect does operate in the manner originally envisioned by Brown and Kodric-Brown [3] and that the converse process (referred to here as the abandon-ship effect) may also be at work. Further work in natural microcosm systems will likely continue to provide important insights into the dynamics of natural populations [33]. 

## Figures and Tables

**Table 1:** Comparison of extinction rates in patches with and without immigration and emigration for *G. wattersoni*. *p*-value is from Fisher’s exact test.

Years	*n*	No Immigration	# Extinctions (%)	Immigration	# Extinctions (%)	*p*
2000–2001	101	63	7 (11.1)	38	2 (5.3)	0.477
2001–2002	89	50	4 (8.0)	39	2 (5.1)	0.692
all	190	113	11 (9.7)	77	4 (5.2)	0.288
**Years**	***n***	**No Emigration**	**# Extinctions (%)**	**Emigration**	**# Extinctions (%)**	***p***
2000–2001	101	57	5 (8.8)	44	5 (11.4)	0.911
2001–2002	89	52	1 (1.9)	37	5 (13.5)	0.078
all	190	109	6 (5.5)	81	10 (12.3)	0.115

## Data Availability

Data from this study are available at: https://doi.org/10.6084/m9.figshare.22304293.
